# Short Term Stability Testing of Efavirenz-Loaded Solid Lipid Nanoparticle (SLN) and Nanostructured Lipid Carrier (NLC) Dispersions

**DOI:** 10.3390/pharmaceutics11080397

**Published:** 2019-08-08

**Authors:** Pedzisai A. Makoni, Kasongo Wa Kasongo, Roderick B. Walker

**Affiliations:** Division of Pharmaceutics, Faculty of Pharmacy, Rhodes University, Grahamstown 6140, South Africa

**Keywords:** efavirenz, solid lipid nanoparticles, nanostructured lipid carriers, stability, Zeta potential, loading capacity, encapsulation efficiency, particle size, polydispersity index, in vitro release

## Abstract

The short term stability of efavirenz-loaded solid lipid nanoparticle and nanostructured lipid carrier dispersions was investigated. Hot High Pressure Homogenization with the capability for scale up production was successfully used to manufacture the nanocarriers without the use of toxic organic solvents for the first time. Glyceryl monostearate and Transcutol^®^ HP were used as the solid and liquid lipids. Tween^®^ 80 was used to stabilize the lipid nanocarriers. A Box-Behnken Design was used to identify the optimum operating and production conditions viz., 1100 bar for 3 cycles for the solid lipid nanoparticles and 1500 bar for 5 cycles for nanostructured lipid carriers. The optimized nanocarriers were predicted to exhibit 10% efavirenz loading with 3% and 4% Tween^®^ 80 for solid lipid nanoparticles and nanostructured lipid carriers, respectively. Characterization of the optimized solid lipid nanoparticle and nanostructured lipid carrier formulations in relation to shape, surface morphology, polymorphism, crystallinity and compatibility revealed stable formulations with particle sizes in the nanometer range had been produced. The nanocarriers had excellent efavirenz loading with the encapsulation efficiency >90%. The optimized nanocarriers exhibited biphasic in vitro release patterns with an initial burst release during the initial 0–3 h followed by sustained release over a 24 h period The colloidal systems showed excellent stability in terms of Zeta potential, particle size, polydispersity index and encapsulation efficiency when stored for 8 weeks at 25 °C/60% RH in comparison to when stored at 40 °C/75% RH. The formulations manufactured using the optimized conditions and composition proved to be physically stable as aqueous dispersions.

## 1. Introduction

Pharmaceutically-relevant colloidal systems consisting of small molecules, with sizes typically ranging between 10 nm and 1 µm, are being used to achieve different therapeutic outcomes. Such carriers include technologies such as liposomes [1], nanoemulsions [2], nanocapsules [3], polymeric nanoparticles [4], solid lipid nanoparticles (SLN) [5,6,7] and nanostructured lipid carriers (NLC) [8,9,10]. A number of different active pharmaceutical ingredients (API) have been incorporated into solid lipid nanoparticles (SLN) and nanostructured lipid carriers (NLC) for oral [11,12], parenteral [13,14], cosmetic or dermatological use [15,16]. Muller et al. [15] and Garud et al. [17] have reported a broad list of API molecules that have been incorporated into SLN and NLC using different manufacturing approaches [17,18]. Efavirenz (EFV) has been incorporated into SLN using solvent emulsification and evaporation [19], modified spontaneous nanoprecipitation [20], homogenization ultrasonication [21] and into NLC using melt emulsification-ultrasonication [22] however most of these techniques use toxic organic solvents such as chloroform, which is unsuitable for human use [23]. The morphology of SLN is dependent on the method used for incorporation of API into the nanoparticles [15,24] and is reliant on a number of factors, including the solubility of the API in the lipid, miscibility of the API and lipid melt, physicochemical properties of solid lipid matrix and the API and the method of manufacture [18].

The major drawbacks of SLN such as API leakage during storage, insufficient drug loading and high water content of SLN dispersions led to the development of NLC [15,25]. API expulsion in SLN has been attributed to polymorphic transitions of solid lipids on storage [26]. Lipid molecules exhibit polymorphism and can therefore transition into consecutive crystalline forms without any alteration in internal structure [27]. Due to the amorphous nature of transitional compounds, they permit incorporation of API which is retained in the lipid matrix. On prolonged storage, the lipid matrices of SLN undergo reversal of polymorphic modification leading to a reduction in the amorphous regions of the matrix due to α and/or *β*’ transitions to the stable *β* form [15,18,24,26], ultimately leading to API expulsion from the SLN.

NLC exhibit similar physicochemical properties to SLN, however they have been developed by nano-structuring the architecture of the lipid matrix in order to increase API loading in addition to preventing drug leakage during storage, thereby resulting in greater flexibility for targeting specific API content and release profiles [24,25]. The structural matrix of NLC is derived from a mixture of solid and liquid lipids that are mixed in specific combination, resulting in a less ordered lipid matrix with numerous imperfections that can accommodate larger amounts of API. NLC particles solidify on cooling but do not recrystallize and therefore remain in an amorphous state [15,24,26]. API expulsion on prolonged storage is less likely to occur from NLC as compared to SLN due to the difference in architecture of the lipid matrices.

Stability testing of pharmaceutical products involves the use of a set of procedures that ultimately aim to ensure that quality, safe and effective products are manufactured [28]. The stability of a formulation in a specific container indicates that the product retains physical, chemical, microbiological and toxicological integrity whilst falling within specifications [28,29]. Consequently, evaluation of the extent to which product specifications are retained throughout a period of storage and use and are similar to those observed at the time of packing, are the goal of stability studies [28,29].

World Health Organization (WHO) [30], International Conference on Harmonization (ICH) [31] and Food and Drug Administration (FDA) [32] guidelines were used to design a stability study protocol that included selection of batches, number of batches, container closure system, sampling frequency, test storage conditions, test specifications, product specifications, test procedure and statistical evaluation of stability data for SLN and NLC. To our knowledge, no reports in which a comparison of formulation and stability of EFV-loaded SLN and NLC manufactured using Hot High Pressure Homogenization (HHPH) have been published. Furthermore, this technique avoids the use of toxic organic solvents and can be readily adapted for scale up production.

**Figure 1:**
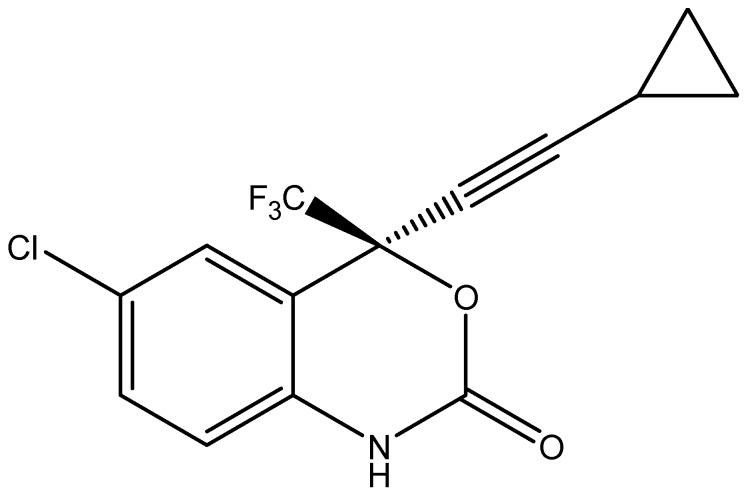
Chemical structure of EFV (C_14_H_9_ClF_3_NO_2_, MW = 315.7) [36].

EFV (Figure 1), is a non-nucleoside reverse transcriptase inhibitor used to treat HIV-1 infected individuals [33,34]. EFV binds selectively to HIV-1 reverse transcriptase (RT) resulting in an allosteric change in RT leading to non-productive binding of incoming nucleotides during DNA polymerization [35]. EFV has low aqueous solubility and high intestinal permeability and is classified as a Biopharmaceutical Classification System (BCS) Class II compound [36]. EFV is effective and requires once-a-day dosing. EFV reaches therapeutic levels in the central nervous system (CNS) and may potentially be used to manage HIV in the CNS [37,38]. However, EFV levels in the CNS are generally high due to “dose-dumping” that is associated with the use of conventional dosage forms and can lead to severe psychiatric side effects. Innovative lipid carriers such SLN and NLC have the potential to sustain and modulate release of EFV into brain tissues and may reduce or limit the incidence of adverse psychiatric effects.

## 2. Materials and Methods

### 2.1. Materials

EFV was donated by Adcock Ingram^®^ Limited (Johannesburg, Gauteng, South Africa). Glyceryl monostearate (GM) and Tween^®^ 80 were purchased from Aspen^®^ Pharmacare (Port Elizabeth, Eastern Cape, South Africa). Transcutol^®^ HP (THP) was donated by Gattefossé SAS (Gattefossé SAS, Saint-Priest Cedex, France). Sodium lauryl sulphate (SLS) was purchased from Sigma Aldrich Chemical Co. (Milwaukee, WI, USA). HPLC-grade water was obtained from a Milli-RO^®^ 15 water purification system (Millipore^®^, Bedford, MA, USA).

### 2.2. Production of EFV-Loaded SLN and NLC Formulations

A discontinuous HHPH technique was used for the production of EFV-loaded SLN and NLC. A total of 5% *w*/*w* lipids was used for all formulations and a 70:30 ratio of GM to THP used for the production of NLC. Briefly the lipid phase containing EFV was heated to approximately 70 °C. An aqueous phase containing Tween^®^ 80 was heated to the same temperature prior to dispersion in the molten lipid phase using a Model T 18 Ultra-Turrax^®^ BS2 homogenizer (Janke & Kunkel GmbH and Co KG, Staufen, Germany) at 1000 rpm for one minute to produce a pre-emulsion. The resultant hot pre-emulsion was then homogenized using an APV 2000 High Pressure Homogenizer (AxFlow Limited, West Sussex, UK) at predetermined pressure and number of homogenization cycles. The nanoemulsion was filled into glass vials and allowed to cool to room temperature (22 °C). All batches were initially characterized on the day of manufacture.

### 2.3. Characterization of SLN and NLC Formulations

#### 2.3.1. Particle Size (PS) and Polydispersity Index (PDI)

The mean PS and PDI of the SLN and NLC was determined with a Nano-ZS Zetasizer (Malvern Instruments Ltd., Worcestershire, UK) using Photon Correlation Spectroscopy (PCS). All samples were diluted with 10 mL HPLC-grade water, to ensure suitable scattering intensity, prior to PCS analysis and placed in a 10 × 10 × 45 mm cell. Measurements were performed in replicate (*n* = 10) at a scattering angle of 90° and temperature of 22 °C. The analysis of PC data was achieved using Mie theory with the real and imaginary refractive indices set at 1.456 and 0.01 respectively.

#### 2.3.2. Zeta Potential (ZP)

The ZP of the nanocarriers was measured using a Nano-ZS Zetasizer (Malvern Instruments Ltd., Worcestershire, UK) set in the Laser Doppler Anemometry (LDA) mode. The sample was prepared as described in Section 2.3.1 and placed into folded capillary cells. All measurements (*n* = 10) were performed at an applied field strength of 20 V/cm and the Helmholtz-Smoluchowsky equation was used in situ to calculate the ZP of each sample.

#### 2.3.3. Transmission Electron Microscopy (TEM)

TEM was used to investigate the shape and surface morphology of the nanoparticles in the original aqueous dispersions. Briefly a drop of the aqueous SLN/NLC dispersion was placed onto a copper grid fitted with a carbon film. The excess liquid was removed using Whatman^®^ 110 hydrophilic filter paper (Whatman^®^ International Ltd., Maidstone, UK) and the sample allowed to dry at room temperature (22 °C) for 24 h. The sample was visualized using a Zeiss Libra^®^ Model 120 TEM (Zeiss, GmbH, Germany).

#### 2.3.4. Scanning Electron Microscopy (SEM)

SEM was used to evaluate the shape and surface morphology of SLN/NLC. Prior to analysis aqueous dispersions of SLN/NLC were lyophilized without a cryoprotectant using a Vacutec freeze drier (Labconco^®^, Corp, Kansas City, MS, USA). The lyophilized sample was mounted onto a carbon stub and sputter-coated with gold under vacuum (0.25 Torr) for 15 min using a sputter-coater (Balzers Union Ltd., Balzers, Lichtenstein). The sample was visualized using a Model TS 5136 LM Vega^®^ Tescan SEM (Tescan, Vega LMU, Czechoslovakia Republic) at a voltage of 20 kV.

#### 2.3.5. Differential Scanning Calorimetry (DSC)

The degree of crystallinity and polymorphism of the nanocarrier systems was determined using a Model Q100 DSC fitted with an RCS 90 refrigerated cooling system (TA instruments, Lukens Drive New Castle, DE, USA). Lyophilized SLN/NLC samples weighing 3–5 mg were heated from 22 °C to 250 °C and subsequently cooled to 22 °C at heating and cooling rates of 10 °C/min. The system was purged with liquid nitrogen at a flow rate of 100 mL/min.

#### 2.3.6. Fourier Transform Infrared Spectroscopy (FT-IR)

The crystalline and polymorphic nature of SLN/NLC was also investigated using FT-IR as a complementary analytical tool to DSC. Lyophilized samples of the nanoparticles were mounted onto a diamond crystal using an applied force of approximately 100 N. The FT-IR spectrum was generated using a FT-IR Spectrum 100 spectrophotometer (Perkin-Elmer^®^ Pty Ltd., Beaconsfield, UK) over the scan range of 4000–650 cm^−1^ at a resolution of 4 cm^−1^.

#### 2.3.7. Loading Capacity (LC) and Encapsulation Efficiency (EE)

The LC and EE of the SLN and NLC for EFV was investigated using a validated reversed-phase high performance liquid chromatography (RP-HPLC) method [39] following filtration of the aqueous dispersion using Centrisart^®^ filter tubes (Satorius AG, Goettingen, Germany). The filter tubes consisted of a filter membrane with a molecular cut-off of 300 KDa at the base of the sample recovery chamber. Approximately 2 mL of an aqueous dispersion of the nanocarriers was placed into the outer chamber of the tube after which the sample recovery chamber was fitted. The unit was allowed to equilibrate for 5 min and centrifuged at 1500 rpm for 10 min using a Model HN-SII IEC centrifuge (Damon, Needham HTS, MA, USA). The amount of EFV in the aqueous filtrate was determined using Equations (1) and (2).

(1) $$LC=\frac{W_{a}-W_{s}}{W_{a}-W_{s\;}+\;W_{L}}\;\times \;100$$

(2) $$EE=\frac{W_{a}-W_{s\;}}{W_{a}}\;\times \;100$$

where *W*_a_ = weight of EFV added to formulation, *W*_s_ = weight of EFV in supernatant after centrifugation, *W*_L_ = weight of lipid added to the formulation.

#### 2.3.8. In Vitro Release

The in vitro release of EFV from SLN and NLC aqueous dispersions was investigated using a Sotax™ CE 7 USP Apparatus 4 (Sotax™ AG, Aesch, Switzerland) equipped with 22.6 mm diameter cells and operated in a closed loop mode at 37 °C. A 5 mm diameter ruby bead was placed at the base of the 22.6 mm sample cell and approximately 2 g of glass beads of 1 mm diameter were added to the base of the conical part of the sample holder, in order to ensure that the liquid sample was retained in the cell. Approximately 6 mL of the aqueous SLN or NLC dispersions were placed into the chamber and a 25 mm diameter glass fiber filter of pore size 0.7 µm (Macherey-Nagel, Düren, Germany) was fitted to the top of the chamber prior to sealing the unit. The dissolution medium used was 2000 mL of a 1% *w/v* SLS solution maintained at 37 °C and was pumped through each cell at a rate of 8.8 mL/min. An aliquot of 1.25 mL of the dissolution medium was withdrawn at specified time intervals and was replaced with fresh medium of similar volume. The in vitro release studies were performed in triplicate and the amount of EFV released from the nanoparticles was quantitated using a validated RP-HPLC method [39].

### 2.4. Stability Studies

The stability of the optimized SLN and NLC formulations was evaluated using Binder^®^ KBF-240 climatic chambers (Binder GmbH^®^ Ltd., Munchen, Germany). The stability studies were undertaken at 25 °C/60% RH and 40 °C/75% RH. The formulations were packed into 100 g glass ointment jars and sealed tightly. The stability was assessed at 0, 1, 2, 4 and 8 weeks following storage. At each pull time the formulation was tested and not returned to the stability chamber. The formulations were assessed in terms of parameters considered benchmarks for establishing stability of colloidal systems and included ZP, PS, PDI, EE and LC.

## 3. Results and Discussion

### 3.1. Production of EFV-loaded SLN Formulations

HHPH was successfully used for the manufacture of EFV-loaded SLN and NLC with Tween^®^ 80. The optimum operating production conditions were established by Box-Behnken Design (BBD) through numerical optimization viz., 1100 bar for 3 cycles for the SLN and 1500 bar for 5 cycles for NLC. The optimized nanocarriers were predicted to have 10% *w*/*w* EFV loaded when 3% and 4% Tween^®^ 80 was used in the SLN and NLC formulations, respectively.

### 3.2. Characterization of SLN and NLC Formulations

#### 3.2.1. Particle Size (PS) and Polydispersity Index (PDI)

The mean PS and PDI of EFV-loaded SLN was 59.00 ± 23.16 nm and 0.382 ± 0.054 measured on the day of manufacture and values of 34.73 ± 0.7709 nm and 0.394 ± 0.027 for mean PS and PDI of EFV-loaded NLC were established on the day of manufacture.

The PDI observed for EFV-NLC was higher than that of the SLN. The substitution of 30% solid lipid with THP decreased the amount of mono, di and acylglycerols in the formulation which tends to broaden the size distribution of NLC [40]. Therefore, when the amount of EFV in SLN and NLC is the same, it is invariable that EFV-loaded NLC would exhibit a larger particle size than EFV-SLN because of the fluidity of THP in the lipid matrix that leads to the expansion of the solid lipid core. However, the size of the nanoparticles may be significantly affected by process variables such as homogenization pressure and number of homogenization cycles used. Different process parameters were used for the manufacture of optimized SLN and NLC the consequence of which are the differences in PS and PDI values observed.

#### 3.2.2. Zeta Potential (ZP)

The optimized EFV-loaded SLN and NLC had a ZP of −32.5 ± 4.99 mV and −22.4 ± 3.72 mV measured on the day of manufacture using HPLC grade water as the dispersion medium. The negative dimension for the ZP is due to the incorporation of Tween^®^ 80 in the formulation. The surfactant is non-ionic and therefore does not ionize, however molecular polarization and adsorption of the surfactant molecule onto the charged water molecules may lead to the formation of an electric double layer that is similar to that observed for ionic surfactants [41,42].

The ZP of EFV-NLC was not as large as that of SLN and was therefore lower than the value that infers adequate stability of these systems (−30 mV). This may be attributed to a shift in the shear plane of the NLC due to the incorporation of liquid lipid into the solid lipid core [41,43] and the formulation was considered unstable. However, it should be acknowledged that reference to 30 mV in order to infer stability only applies to colloidal systems that have been stabilized using electrostatic interactions only. Tween^®^ 80 imparts steric stability to the nanoparticles in addition to electrostatic repulsion [41,43] consequently the ZP of NLC is adequate for the purpose of providing stability to nanoparticles.

#### 3.2.3. Transmission Electron Microscopy (TEM)

**Figure 2:**
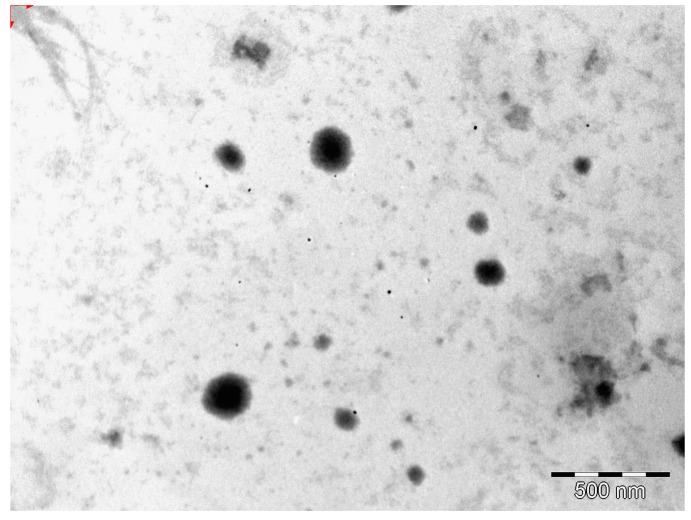
TEM micrograph of optimized EFV-loaded SLN.

**Figure 3:**
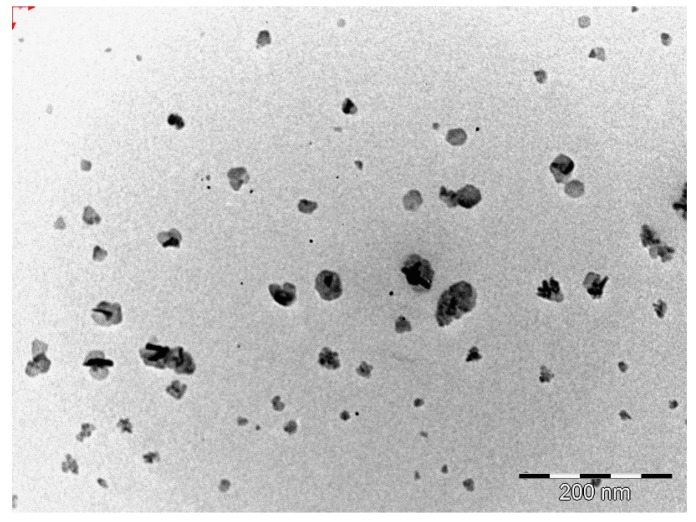
TEM micrograph of optimized EFV-loaded NLC.

The morphology of the optimized EFV-loaded SLN and NLC products in the original aqueous dispersion was investigated using TEM and the data generated are depicted in Figure 2 and Figure 3.

The EFV-SLN particles are mainly present as discrete entities when compared to EFV-NLC particles. The SLN particles appear to be larger in size when compared to the NLC particles that is consistent with the lower PS and higher PDI observed for NLC.

The shape of the SLN and/or NLC particles may be determined by the purity of the lipid used. Lipids that are chemically pure and homogenous usually produce nanoparticles that appear cuboid in shape [6]. Similarly, spherical particles are usually observed with the use of lipids that are chemically poly-disperse [6,41,44]. Therefore, it was expected that EFV-loaded SLN would be cuboidal in shape when compared to the more spherical NLC observed. However, the inclusion of a surfactant in the SLN formulation has the potential to distort the crystal structure of pure lipids leading to a change in morphology of the nanoparticles from cuboid to spherical [6]. Therefore, the SLN and NLC produced in these experiments were generally spherical in shape.

#### 3.2.4. Scanning Electron Microscopy (SEM)

**Figure 4:**
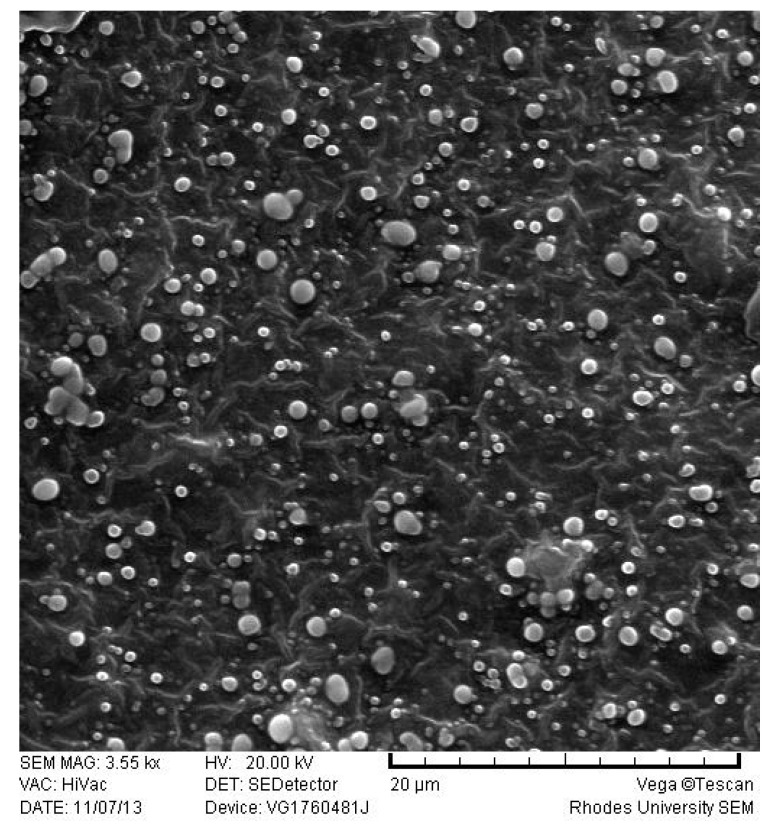
SEM micrograph of optimized EFV-loaded SLN.

**Figure 5:**
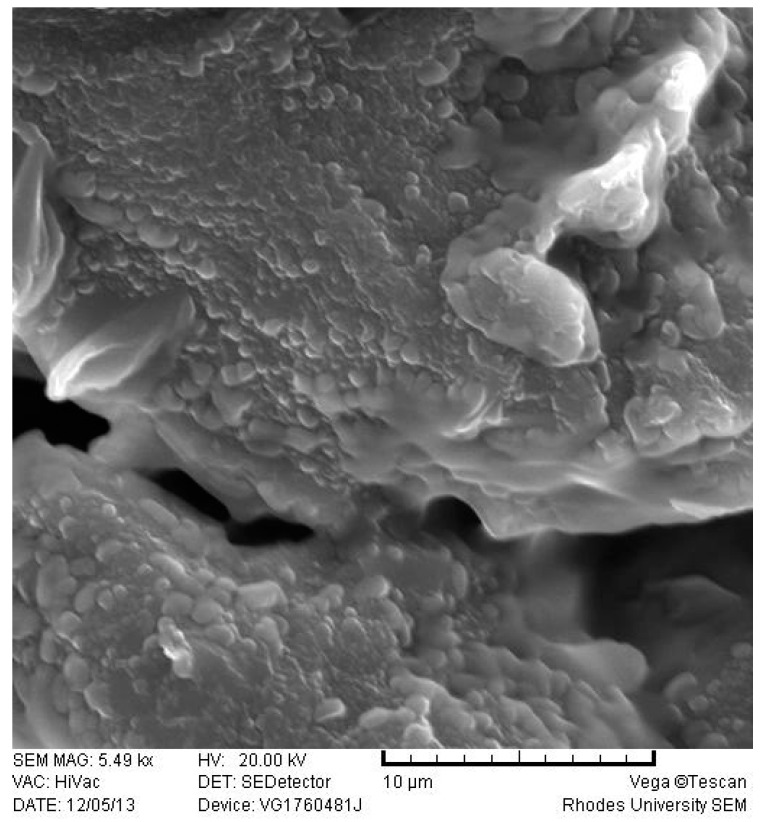
SEM micrograph of optimized EFV-loaded NLC.

The shape and surface morphology of the optimized EFV-SLN and EFV-NLC following lyophilization were evaluated using SEM. These data are depicted in Figure 4 and Figure 5.

EFV-loaded SLN and NLC appeared smooth and somewhat spherical. However, the surface of the NLC appeared to be smoother than that of the SLN possibly due to the presence of THP in the lipid matrix. The inclusion of THP in the lipid phase to produce NLC imparted some variation to the surface morphology of the particles. The SEM micrographs also reveal the presence of micro-particles. These observations differ to PCS data. It has been suggested that lyophilization of aqueous dispersions of SLN or NLC may promote aggregation of nanoparticles especially when freeze drying without the use of a cryoprotectant is undertaken [45,46]. The aggregates may appear as micro-particles when visualized with SEM.

#### 3.2.5. Differential Scanning Calorimetry (DSC)

**Figure 6:**
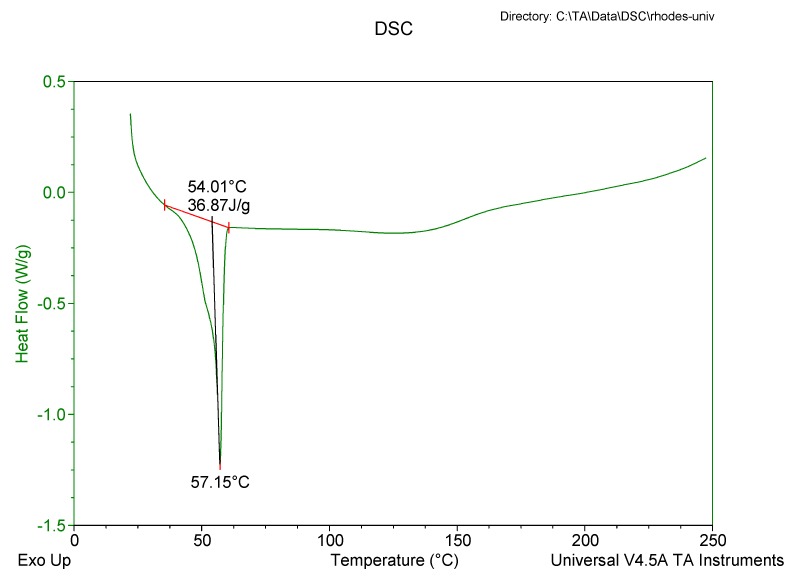
DSC thermogram for optimized EFV-loaded SLN.

**Figure 7:**
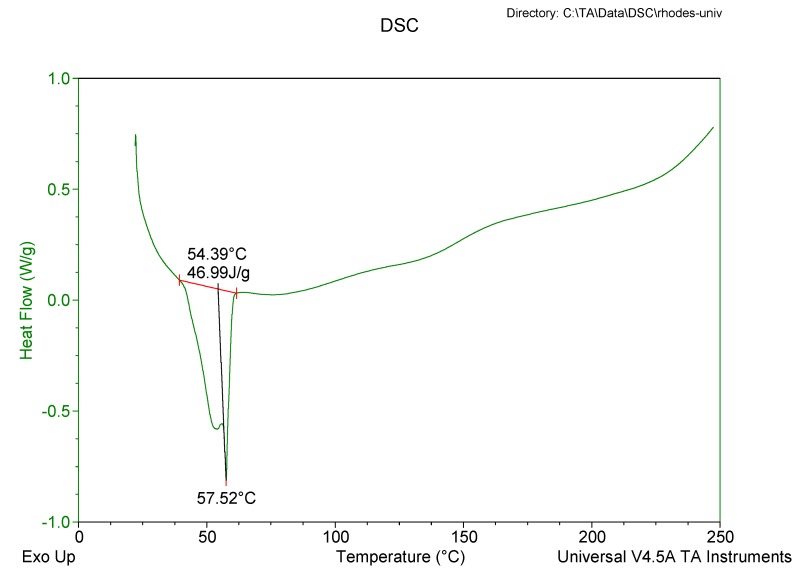
DSC thermogram for optimized EFV-loaded NLC.

**Figure 8:**
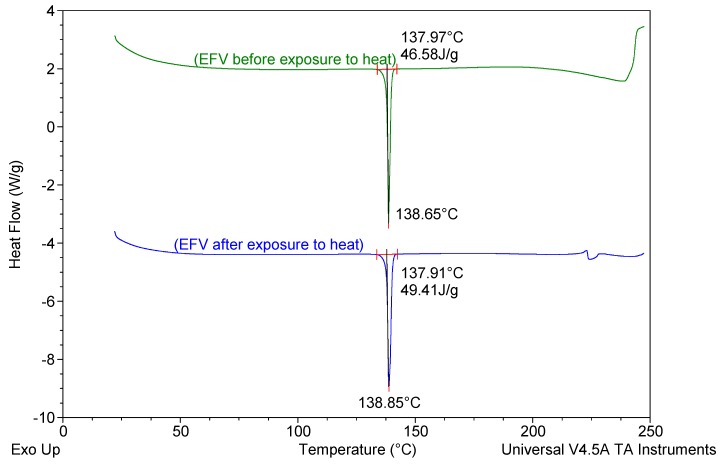
DSC thermograms of EFV prior to and following exposure to a temperature of 70 °C for 1 h.

**Figure 9:**
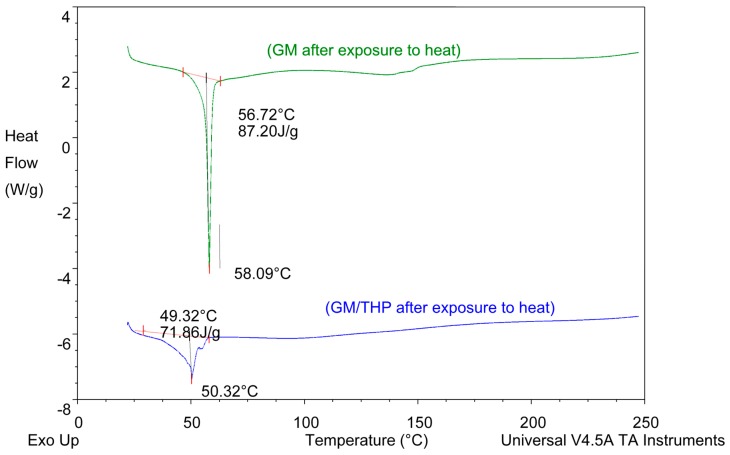
DSC thermograms of GM and of a binary mixture of GM and THP generated following exposure to 70 °C for 1 h.

DSC was used to evaluate the polymorphism of optimized EFV-loaded SLN and NLC, and the data generated are depicted in Figure 6 and Figure 7. Figure 8 and Figure 9 depict DSC thermograms of EFV and the lipids used in the preparation of the nanocarriers for comparative purposes. The thermograms were developed prior to and after exposure of the nanocarriers to 70 °C so as to mimic the manufacturing process using HHPH.

The DSC thermogram for EFV-loaded SLN revealed the presence of a single endothermic event and the absence of a peak for EFV indicating the existence of a single polymorphic form of the solid lipid and that the EFV was molecularly dispersed in the SLN matrix. The onset and melting temperature at 54.01 °C and 57.15 °C respectively are consistent with the presence of the α-polymorphic form of GM. Similarly, the DSC thermogram of EFV-loaded NLC revealed that EFV was molecularly dispersed in the NLC however, the presence of two endothermic events implied the co-existence of α- and β’-polymorphic forms of the lipids. In contrast to Figure 9, the β’-polymorphic form is more dominant than the α-modification in the NLC as can be clearly observed from the intensities of two peaks that could be attributed to a change in the lattice structure of the lipid mixture due to incorporation of EFV and Tween^®^ 80 in the binary mixture of the lipid used for the formulation of EFV-loaded NLC.

#### 3.2.6. Fourier Transform Infrared Spectroscopy (FT-IR)

**Figure 10:**
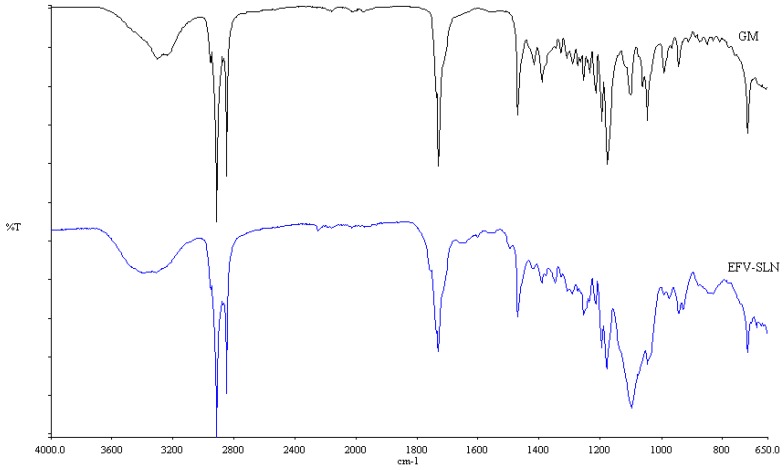
FT-IR spectra of optimized EFV-loaded SLN and GM following exposure to heat.

**Figure 11:**
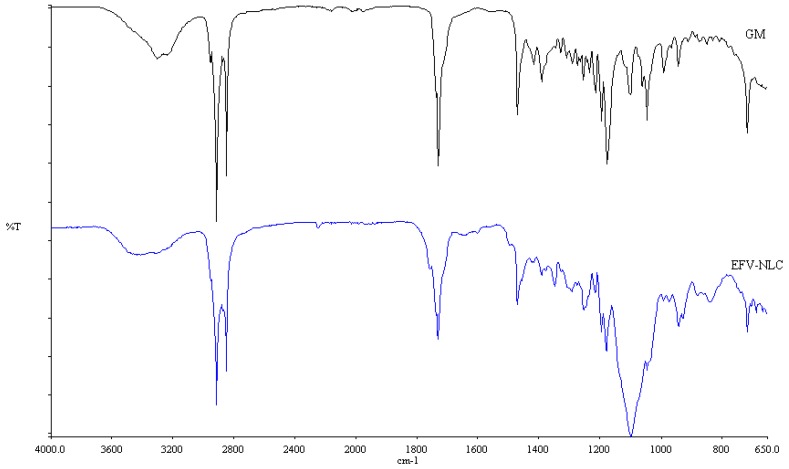
FT-IR spectra of optimized EFV-loaded NLC and GM after exposure to heat.

FT-IR was used to assess the crystalline nature of the optimized EFV-loaded SLN and NLC, and the spectra generated are depicted in Figure 10 and Figure 11. These data also show the FT-IR spectra of GM that was generated following exposure to heat for comparative purposes to facilitate data interpretation.

The FT-IR spectra for the colloidal systems reveal the presence of the characteristic bands of the solid lipid material, GM at a frequency between 2950 and 2910 cm^−1^, corresponding to a C–H stretch. However, the characteristic bands for EFV at a frequency of 3311 cm^−1^ corresponding to a N–H stretch, 2250 cm^−1^ due to the carbon to carbon triple bond stretch, 1749 cm^−1^ for the carbonyl stretch, 1602 cm^−1^ due to carbon to carbon double bond stretching, between 1350–1120 cm^−1^ for the CF_3_ stretch, between 1096–1098 cm^−1^ for the C–Cl bond stretching and between 900–650 cm^−1^ which is due to aromatic ring detection were not observed for both SLN and NLC confirming the DSC results suggesting that EFV was molecularly dispersed in the nanoparticles. Furthermore, no additional signals due to the incorporation of THP in the NLC formulation were observed suggesting no interaction occurred between the liquid and solid lipids used. The band intensity of SLN looks similar to that of GM suggesting that the crystalline nature of the lipid did not change to any great extent. However, the NLC band intensity is low indicating the presence of amorphous material in the carriers due to the inclusion of THP.

#### 3.2.7. Loading Capacity (LC) and Encapsulation Efficiency (EE)

The LC and EE value of the optimized SLN for EFV determined on the day of manufacture were 9.68 ± 1.77% and 96.77 ± 0.45% respectively. Similarly, 9.995 ± 0.67%, and 99.93 ± 0.413% were the LC and EE for NLC. The SLN exhibited relatively low LC and EE values for EFV compared to those observed for NLC formulations that may be due to the relatively high solubility of EFV in liquid lipid when compared to the solubility of EFV in the solid lipid alone. It has previously been shown that high LC and EE values can be observed when the solubility of an API is increased in the lipid used [18,47]. In addition, the addition of THP to GM may increase the complexity of the internal solid lipid structure and this disordered molecular arrangement may create additional voids for entrapping the API [24,25]. Regardless of the relatively small differences in the magnitude of the values for LC and EE for both SLN and NLC, the EFV payload observed in these studies was considered adequate.

#### 3.2.8. In Vitro Release

**Figure 12:**
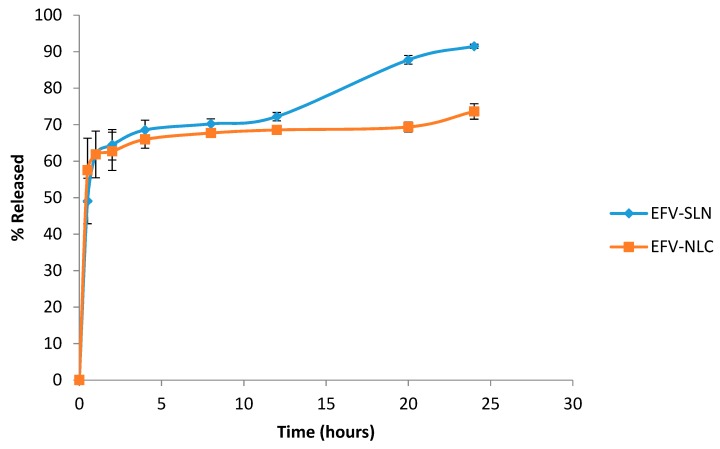
In vitro release profile of EFV from the optimized SLN and NLC formulations (*n* = 3).

The in vitro release profile of EFV from the optimized SLN and NLC formulations are depicted in Figure 12.

The in vitro release data show that the cumulative amount EFV released over a 24 h period was higher from SLN (91.5 ± 3.423%) than from NLC (73.6 ± 4.34%) possibly due to the high degree of solubility of EFV in the lipid matrix of the NLC and the complex architecture of the lipid matrix that reduces the expulsion of EFV from the carrier into the dissolution medium. The SLN appear to release EFV readily as the solid matrix is more crystalline and well-ordered allowing EFV to escape relatively easily. The data reveal that EFV release from the SLN and NLC is biphasic with an initial burst release followed by slower and more sustained release. The initial burst was observed between 0–3 h and suggests the presence of an EFV-rich region in the exterior lipid layer of the nanoparticles. The presence of high levels of EFV in the exterior lipid has been shown to be due to the addition of surfactant directly into the aqueous phase and the use of HHPH for production, leading to the formation of a surfactant-lipid boundary layer enriched with API [18,24]. Following the initial burst release the nanoparticles released EFV in a sustained release manner that can be attributed to the shear stress of agitation during dissolution testing which ultimately diminishes the surfactant layer on the surface of the nanoparticles, leading to sustained release of EFV from the decomposition of the integrated lipid core structure of the SLN and NLC [47,48].

Mathematical modelling of the in vitro release data revealed that the release of EFV from SLN followed first order release kinetics exhibiting an *R*^2^ value of 0.9667 implying that release of EFV from SLN is proportional to the amount of EFV remaining in the nanoparticles and that release diminishes over time. However, EFV release from the NLC followed Higuchi release with an *R*^2^ value of 0.9236 implying diffusion controlled release from the NLC occurs after the initial burst release.

### 3.3. Stability Testing

#### 3.3.1. Particle Size and Size Distribution

**Figure 13:**
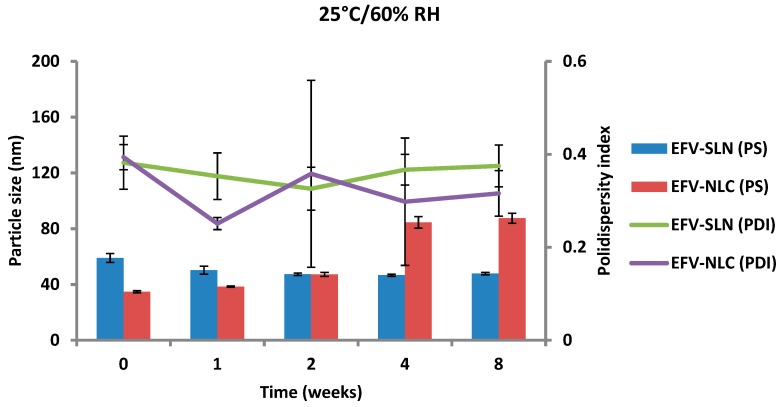
Particle size and size distribution of SLN and NLC following storage at 25 °C/60% RH.

**Figure 14:**
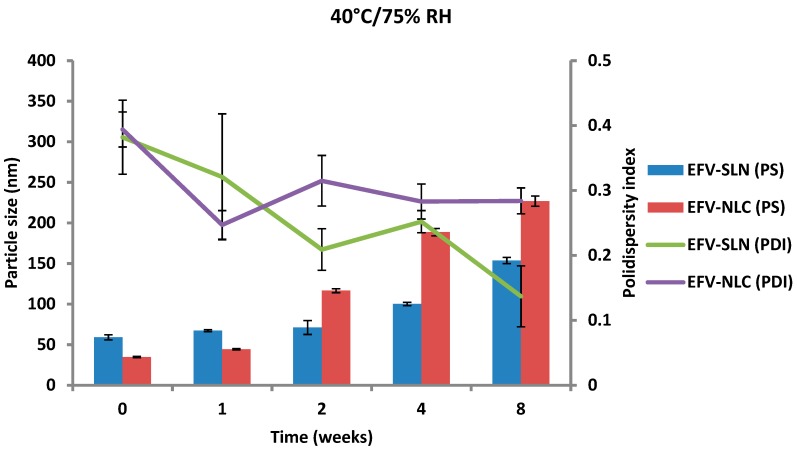
Particle size and size distribution of SLN and NLC following storage at 40 °C/75% RH.

The PS and PDI of the nanoparticles generated following storage at 25 °C/60% RH and 40 °C/75% RH are depicted in Figure 13 and Figure 14.

The PS of the optimized EFV-loaded SLN and NLC stored at 25 °C/60% RH ranged between 46.64 and 59.0 nm and between 34.73 to 87.47 nm, respectively and the PS of the optimized EFV-loaded SLN and NLC stored at 40 °C/75% RH ranged between 59.0 and 153.7 nm and between 34.73 to 226.9 nm, respectively. In general, the PS of SLN and NLC increased during storage, apart from the EFV-loaded SLN stored at 25 °C/60% RH that maintained a fairly constant size. Particle growth was slower when the nanoparticles were stored at 25 °C/60% RH, as at higher temperatures (40 °C/75% RH) the kinetic energy of the system increases leading to increased collisions between particles, the consequence of which is aggregation and an increase in the size of the particles [25,49,50]. In addition, a high level of the film emulsifier has been shown to avert fusion of film layers after particle collision [49]. Microviscosity which is the friction experienced by particles, is a temperature dependent factor and decreases with an increase in temperature leading to particle destabilization and possibly agglomeration [49,51].

The increase in particle growth is correlated with the decrease in the ZP. It was observed that the rate of particle growth for NLC under both storage conditions was higher than that observed for SLN that can be attributed to the presence of a liquid component inside the lipid matrix of this carrier which might cause aggregation of the particles [25]. However, PCS data reveal that the size of all particles remained within the nanometer range following storage for 8 weeks. In addition, the low and constant PDI values observed for all formulations reveal that the SLN and NLC particles were physically stable in terms of PS and PDI at these storage conditions for at least 8 weeks.

#### 3.3.2. Zeta Potential (ZP)

**Figure 15:**
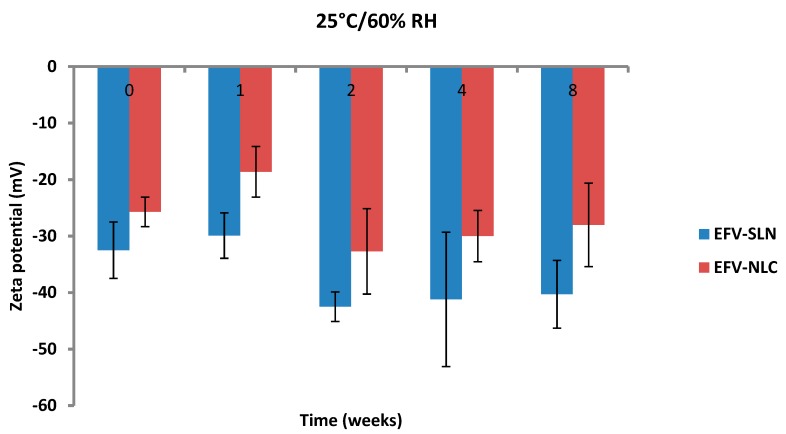
Zeta potential of SLN and NLC following storage at 25 °C/60% RH.

**Figure 16:**
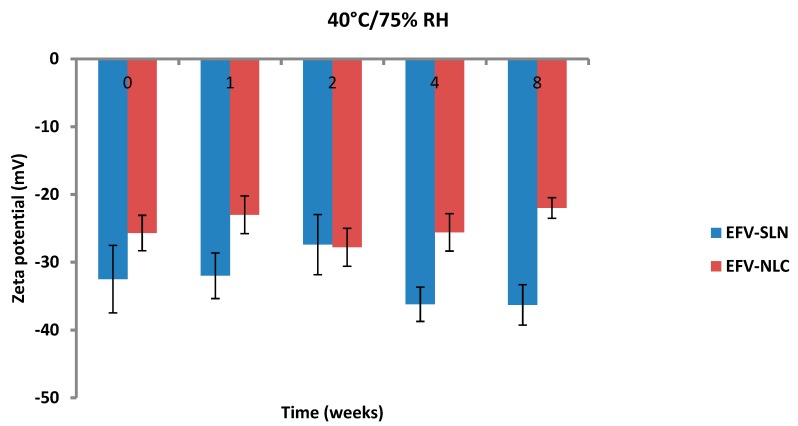
Zeta potential of SLN and NLC following storage at 40 °C/75% RH.

The ZP of EFV-loaded SLN and NLC following storage at 25 °C/60% RH and 40 °C/75% RH is depicted in Figure 15 and Figure 16.

The ZP of the optimized EFV-loaded SLN and NLC stored at 25 °C/60% RH ranged between −29.9 to −42.5 mV and −18.6 to −32.7 mV, respectively. Similarly, the ZP for the optimized EFV-loaded SLN and NLC stored at 40 °C/75% RH ranged between −27.4 to −36.3 mV and −22.0 to −27.8 mV, respectively. The ZP for SLN indicate good physical stability of the nanoparticles (≤−30 mV) following storage at 25 °C/60% and 40 °C/75% for a period of 8 weeks. Although the values for NLC under similar storage conditions were lower, the stability of the nanoparticles can be inferred since the presence of polysorbate provides stability through both electrostatic and steric hindrance mechanisms.

The ZP for all formulations generally decreased from the day of manufacture up to 1 and 2 weeks of storage after which the ZP was constant for up to 8 weeks. The increase in stability of the formulations could be attributed to the use of HHPH that leads to the formation of a surfactant rich interface [18,24] on the surfaces of the particles. The increase in ZP after 1 to 2 weeks of storage may be attributed to the immediate crystalline re-orientation of the formulations on storage resulting in changes in the surface charges of the particles. In addition, different sides of a crystal can acquire a different charge density, as is the case with aluminium silicates such as bentonite [52]. Long β crystals can be formed during one-dimensional crystal growth ultimately resulting in modification of the surface ratio of differently charged crystal sides and consequently changes and/or variations in the ZP [52]. The constant ZP observed thereafter suggests no alteration to the surface properties of the particles of the SLN and NLC for the 8 week period of storage.

The storage of the nanoparticles at 40 °C/75% RH led to a faster increase in the ZP when compared to data for samples stored at 25 °C/60% RH, suggesting they were less stable. Following 8 weeks of storage, the ZP for SLN at 25 °C/60% RH was −40.3 mV and that for SLN stored at 40 °C/75% RH was −36.3 mV. In addition, the ZP for NLC was −28 mV and −22 mV following storage at 25 °C/60% RH and 40 °C/75% RH for 8 weeks, respectively. Generally, the ZP increased with an increase in energy input that has been reported to lead to changes in the crystalline structure of lipids [53]. This crystal re-orientation can ultimately lead to changes of the charge on the surface(s) of the particle and consequently a change in the ZP [49]. However, Riddick [54] defined the agglomeration threshold in dispersions with a ZP range of −15 to −10 mV which is similar to the data generated in these studies as no agglomeration of formulations was observed following storage for 8 weeks.

#### 3.3.3. Encapsulation Efficiency (EE) and Loading Capacity (LC)

**Figure 17:**
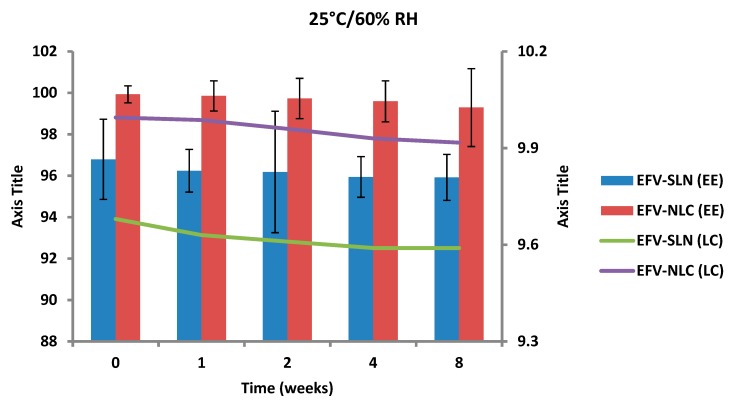
Encapsulation efficiency and loading capacity of SLN and NLC following storage at 25 °C/60% RH.

**Figure 18:**
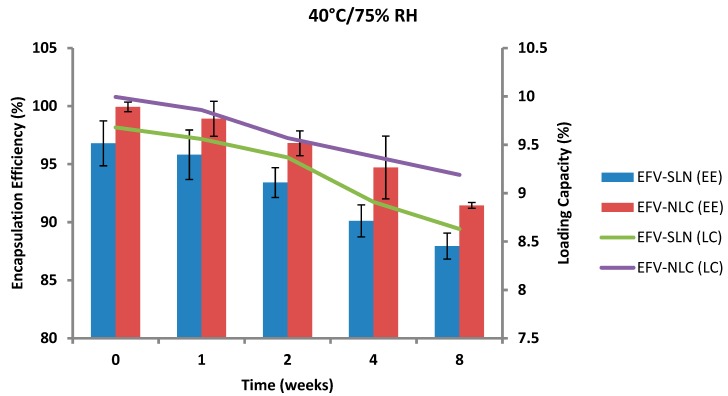
Encapsulation efficiency and loading capacity of SLN and NLC following storage at 40 °C/75% RH.

The EE and LC of optimized EFV-loaded SLN and NLC following storage at 25 °C/60% RH and 40 °C/75% RH are depicted in Figure 17 and Figure 18.

The EE for the optimized EFV-loaded SLN and NLC stored at 25 °C/60% RH ranged between 96.79–95.92% and between 99.93–98.81%, respectively. The corresponding LC for SLN and NLC ranged between 9.68–9.59% and between 9.99–9.88%, respectively. The EE for the optimized EFV-loaded SLN and NLC stored at 40 °C/75% RH ranged between 96.79–87.94% and 99.93–91.44% respectively. The corresponding LC for SLN and NLC ranged between 9.68–8.63% and 9.99–9.19% respectively. Generally, the NLC exhibited higher EE than the SLN over the 8 week period. EFV expulsion was also lower for NLC particles in comparison to that observed for SLN particles. This phenomenon could be attributed to the inclusion of liquid lipid in the solid lipid leading to a disturbance of the crystal structure of the solid lipid and the creation of imperfections in the crystal lattice creating additional space to accommodate more EFV, thereby improving EE and reducing API expulsion [24,25].

The degree of crystallinity of SLN and NLC was calculated from the ratio of SLN and NLC enthalpy to that of the bulk lipid [55]. The degree of crystallinity generated for SLN and NLC were 67% and 65%, respectively. The NLC had a slightly lower degree of crystallinity in comparison to SLN implying a relatively high degree of imperfection in the crystal lattice allowing for increased inclusion of EFV in the NLC.

The amount of EFV entrapped in SLN and NLC remained constant over the 8 week storage period at 25 °C/60% RH. However, at the higher temperature 40 °C/75% RH, the EE and LC of the nanocarriers revealed a significant decrease that can be correlated to the polymorphic state of the lipid. Following production of particles using HHPH the lipid in SLN is present in the α form and that of the NLC co-exists in the α and β’ forms. The input of high temperatures such as 40 °C/75% RH facilitates the conversion of lipids to different polymorphic forms during storage leading to increased EFV expulsion. Freitas and Müller [53] have reported the transformation of SLN from the β’ form accompanied by gel formation following the input of kinetic energy on storage at 50 °C. In addition, Das et al. [8] reported a decrease in EE values of SLN and NLC stored at 25 °C when compared to those stored at between 2–8 °C.

## 4. Conclusions

A hot high pressure homogenization method was successfully used for the manufacture of efavirenz-loaded solid lipid nanoparticles (SLN) and nanostructured lipid carriers (NLC). Tween^®^ 80 was used to stabilize the lipid nanocarriers. A Box-Behnken Design was used to deduce the optimum operating production conditions viz., 1100 bar for 3 cycles for the SLN and 1500 bar for 5 cycles for NLC. The size of the optimized SLN and NLC formulations was in the nano range and exhibited excellent loading capacity for efavirenz with an encapsulation efficiency >90%. However, SLN had a lower encapsulation efficiency and loading capacity when compared to NLC. The optimized SLN and NLC exhibited biphasic in vitro release patterns with burst release during the initial 3 h followed by sustained release over the remaining period of the 24 h release run. The release of EFV from the optimized SLN and NLC followed first order and Higuchi release kinetics, respectively suggesting that NLC would be the most suitable carriers for EFV with the potential to sustain or control the release of EFV. Both systems exhibited excellent stability in terms of Zeta potential, particle size, polydispersity index and encapsulation efficiency following storage at 25 °C/60% RH for eight weeks. However, the delivery systems were relatively unstable when stored at 40 °C/75% RH. Consequently, approaches to improve the physical stability of EFV-loaded SLN and NLC must be investigated to produce colloidal carrier systems suitable for pharmaceutical and/or therapeutic manipulation with the potential to sustain EFV release into brain tissue so as to reduce or limit the incidence of adverse psychiatric effects of the molecule.
